# Expanded G4C2 repeats linked to *C9ORF72* ALS and FTD form length-dependent RNA foci, sequester RNA binding proteins and are neurotoxic

**DOI:** 10.1186/1750-1326-8-S1-O14

**Published:** 2013-09-13

**Authors:** Youn-Bok Lee, Han-Jou Chen, João N Peres, Jorge Gomez, Valentina Sardone, Agnes L Nishimura, Emma Scotter, Caroline Vance, Maja Štalekar, Yoshitsugu Adachi, Claire Troakes, Jack Miller, Bradley Smith, Frank Hirth, Boris Rogelj, Corinne Houart, Christopher E Shaw

**Affiliations:** 1 Department of Clinical Neuroscience, Institute of Psychiatry, London UK; 2 MRC Centre for Developmental Neurobiology, King’s College London, London SE5 8AF, UK; 3 Department of Biotechnology, Jožef Stefan Institute, Jamova 39, SI-1000 Ljubljana, Slovenia; 4 Department of Neuroscience, Institute of Psychiatry, London, UK

## Background

The GGGGCC (G4C2) intronic repeat expansion within *C9ORF72* is the most common genetic cause of amyotrophic lateral sclerosis (ALS) and frontotemporal dementia (FTD) [1,2]. The mechanism by which the G4C2 intronic repeats cause neurodegeneration is unknown. Decreased tissue levels of the *C9ORF72* transcript implicate a loss of protein function due to haploinsufficiency, intranuclear neuronal RNA foci have been observed in ALS and FTD tissues, suggesting that G4C2 RNA may be toxic [1].

## Materials and methods

In order to determine whether expanded G4C2 transcripts might be toxic and sequester RNA binding proteins we generated G4C2 repeats with 8x, 38x and 72x and expressed them in a range of neuronal and non-neuronal cell lines, primary neurons and zebrafish embryos. We used fluorescent *in situ* hybridisation probes for G4C2 to detect RNA foci in transfected cells and human brain tissue form C9ORF72 mutation carriers. We assessed the toxicity by cell counting, activated caspase, PARP cleavage and Annexin V expression transfected cells and by TUNEL assay in zebrafish embryos. We probed transfected cells with antibodies to 30 RNA binding proteins and then tested the best candidates by immunohistochemistry in human brain tissues. We used biotinylated G4C2 x72 probes to pull down repeat binding proteins and confirmed binding by western blot.

## Results

Cellular toxicity was associated with the nuclear retention of transcripts containing 38x and 72x repeats and the appearance of RNA foci. By placing the repeats 3’ to EGFP we were able to monitor nuclear export and show that transfected neuroblastoma cells, but not HEK cells, declined in number over time due to apoptotic cell death. This was most marked in those showing the greatest nuclear retention and foci, indicating cell type and neuron-specific toxicity of longer G4C2 repeats.

In order to explore the sequestration hypothesis we screened antibodies to 30 RNA binding proteins on 72x transfected neuroblastoma cells and demonstrated colocalization of G4C2 RNA foci with three proteins. Using biotinylated 72x RNA we were only able to pull down one of these indicating that it is able to bind G4C2 RNA directly. On probing human cerebellar ALS and FTD tissues we detected a striking colocalization of this protein with 70% of RNA foci.

## Conclusions

Our observation that longer stretches of G4C2 RNA form neurotoxic foci and bind specific RNA binding proteins is similar to other intronic microsatellite expansion disorders where CUG (DM1) and CCUG (DM2) intronic repeats generate RNA foci in cells, animal models and in patients with myotonic dystrophy.
